# Fatty Acid and Related Potassium Kv2 Channel Blockers: Toxicity and Physiological Actions on Mosquitoes

**DOI:** 10.3390/insects9040155

**Published:** 2018-11-01

**Authors:** Fabien Démares, Quentin Coquerel, Gary Richoux, Kenneth Linthicum, Jeffrey Bloomquist

**Affiliations:** 1 Neurotoxicology Laboratory, Department of Entomology and Nematology, Emerging Pathogens Institute, University of Florida, Gainesville, FL 32610, USA; quentin.coquerel@ufl.edu (Q.C.); grichoux@ufl.edu (G.R.); 2 USDA, ARS, Center for Medical, Agricultural and Veterinary Entomology, Gainesville, FL 32608, USA; Kenneth.Linthicum@ars.usda.gov

**Keywords:** fatty acids, potassium channel, patch-clamp, toxicity, *Anopheles gambiae*

## Abstract

Potassium channels constitute a very diverse group involved in neural signaling, neuronal activity, membrane potential maintenance, and action potential generation. Here, we tested the mammalian potassium channel blockers TRAM-34 and 5-hydroxydecanoate (5-HDC), as well as certain fatty acids (FA) that might fit in the lumen of the pore and block channel activity by obstructing K^+^ ion passage. Kv channel blockers could be leads for a novel pesticide type. Insecticidal activity was assessed by topical application to *Anopheles gambiae* adult mosquitoes, paralysis in a headless larval assay, at the cellular level with patch-clamp recordings of engineered HEK cells expressing *Ag*Kv2.1 channels, as well as central nervous system recordings from larval *Drosophila* *melanogaster*. With only one hydroxyl group difference, decanoic acid had a consistently greater effect than 5-HDC in blocking Kv channels, paralyzing larvae, and killing mosquitoes. The 11-dansylamino undecanoic acid (DAUDA) blockage of eukaryotic Kv channels is demonstrated for the first time, but it failed to kill adult mosquitoes. We synthesized alkyl esters from DAUDA and decanoic acid in an effort to improve cuticular penetration, but it had little impact upon adult toxicity. TRAM-34 and rolipram did not show activity on Kv channels nor potent insecticidal effect on adult mosquitoes. Furthermore, co-application of test compounds with permethrin did not increase mortality in adults. In conclusion, the compounds tested had modest insecticidal and synergistic activity.

## 1. Introduction

The ion channels are important macromolecular structures and are major actors in osmotic balance and cell signaling. There is great diversity in these channels with regard to their expression profile, location within cells, ion permeability, and voltage-dependence [1]. The voltage-gated ion channels are involved in many signaling processes, especially action potential conduction. Each phase of the action potential is characterized by different ion permeability and movement, which underlies a different voltage-gated ion channel [2]. The initial depolarizing phase starts with the opening of voltage-gated Na^+^ channels, triggering an inward flow of sodium ion current that rapidly inactivates. Repolarization is achieved by the opening of voltage-gated K^+^ channels, which release an outward flow of potassium ions out of the cell. In terms of structure and composition, sodium and potassium channels share some analogy: each homology domain has six transmembrane segments, S1 to S4, that include the voltage sensor and segments S5–S6 form the pore of the ion channel [2]. One important difference though, one sodium channel subunit comprises four domains, when one potassium channel subunit is only made of one. Thus, a functional voltage-gated K^+^ channel requires the assembly of four subunits and, together, can form homo- or hetero-tetramers [3,4].

Mutants of the gene coding for voltage-gated potassium channels in *Drosophila melanogaster* led to the identification of several subfamilies, namely *Shaker*, *Shab*, *Shaw,* and *Shal*, that correspond to Kv1, Kv2, Kv3, and Kv4 channels, respectively [5,6]. In this report, we focus on one Shab member, the Kv2.1 channel. Due to their electrophysiological properties, i.e., voltage-dependence profile and slow inactivation, Kv2 channels are often described as “delayed rectifier”. Different compounds can block the activity of these channels, with variable potency and efficacy, such as 4-aminopyridine, quinidine [7] or hanatoxin [8]. The latter compound is a spider toxin which acts by occlusion of the pore [9].

It has been previously established that (a) polyunsaturated fatty acids (FA) can block delayed-rectifier potassium channels [10,11], and that (b) voltage-gated Kv2 channels of mosquitoes could represent interesting targets for developing new pesticidal compounds [12,13]. A potential issue with targeting potassium channels is specificity of action due to the wide variety of channel types, as well as selectivity as it pertains to non-target organisms. As the main vector of malaria, *Anopheles gambiae* is the target of several control strategies, especially involving the use of pyrethroids [14], but growing concerns have been expressed regarding the increasing insecticide resistance of *Anopheles* [14,15], calling for another approach and possibly new biopesticides.

In this work, we tested fatty acid compounds as potential insecticides against *Anopheles gambiae* mosquitoes, from channel block under patch-clamp conditions to larval and adult toxicity. The fatty acids tested were 5-hydroxydecanoate (5-HDC), decanoic acid, and 11-dansylamino undecanoic acid (DAUDA). Interestingly, 5-HDC blocks the mitochondrial ATP-sensitive K^+^ channels [16], but it has never been tested on voltage-gated K^+^ channels. Similarly, DAUDA has been shown to bind inside the pore of the prokaryotic K^+^ channel KcsA [17], an evolutionarily close relative to eukaryotic voltage-gated K^+^ channels. Two other compounds investigated in this study were rolipram and TRAM-34, which have been identified as K^+^ channel activity modulators [18,19,20,21]. In the present report, these compounds have been tested as putative insecticides. Also, another strategy to improve the insecticidal activity of the compounds is to look at the synergistic effect with a common pyrethroid, permethrin. The synergistic action of pyrethroids with organophosphates have been documented [22,23] and tested on insects [24]. Synergistic action of propoxur [25] and flonicamid [26] with permethrin has been demonstrated on mosquitoes, so we further examined the utility of K^+^ channel blockers as permethrin synergists for control of disease-vectoring mosquitoes. In light of these results, we also discuss the use of fatty acids as biochemicals in the scope of integrated pest management, to better fit the legislative framework of pesticides regulation [27].

## 2. Materials and Methods

### 2.1. Chemicals and Reagents

Commercially available compounds were purchased from Sigma-Aldrich (St. Louis, MO, USA), including DAUDA, decanoic acid, L-aspartic acid, permethrin, and rolipram; and from Enzo Life Sciences (Farmingdale, NY, USA), for compounds 5-hydroxydecanoate (5-HDC) and TRAM-34. We also tested esterified forms of FAs: ethyl undecanoate, purchased from Sigma-Aldrich, as well as (1) methyl DAUDA, and (2) methyl decanoate, which were synthetized according to the following procedure, adapted from Okano and colleagues [28]. For (1), thionyl chloride (11.7 µL, 0.16 mmol, 1.64 g/mL, 1.4 equiv) was added dropwise to a stirred solution of DAUDA (50 mg, 0.115 mmol, 1 equiv) in methanol (5 mL/mmol) at 0 °C. The mixture was stirred for 2 h, after which time the TLC indicated the reaction was complete. The reaction mixture was then concentrated under reduced pressure. Purification by silica gel column chromatography afforded the product as a white solid (16 mg, 32%). ^1^H NMR (500 MHz, Chloroform-d) δ 8.60 (d, *J* = 8.5 Hz, 1H), 8.34 (d, *J* = 8.6 Hz, 1H), 8.28 (d, *J* = 7.3 Hz, 1H), 7.63–7.54 (m, 2H), 7.24 (d, *J* = 7.6 Hz, 1H), 4.59 (d, *J* = 6.8 Hz, 1H), 3.69 (s, 3H), 2.95 (s, 6H), 2.91 (q, *J* = 6.8 Hz, 2H), 2.31 (t, *J* = 7.5 Hz, 2H), 1.62 (p, *J* = 7.4 Hz, 2H), 1.37 (m, 4H), 1.21–1.06 (m, 10H). For (2), thionyl chloride (147.3 µL, 2.03 mmol, 1.64 g/mL, 1.4 equiv) was added dropwise to a stirred solution of decanoic acid (250 mg, 1.45 mmol, 1 equiv) in methanol (5 mL/mmol) at 0 °C. The mixture was stirred for 2 h, after which time TLC indicated the reaction was complete. The reaction mixture was then concentrated under reduced pressure. Purification by silica gel column chromatography afforded the product as a white solid (270 mg, > 99%). ^1^H NMR (500 MHz, Chloroform-d) δ 3.66 (s, 3H), 2.29 (t, *J* = 7.6 Hz, 2H), 1.62 (p, *J* = 7.3 Hz, 2H), 1.27 (d, *J* = 14.9 Hz, 12H), 0.87 (t, *J* = 6.3 Hz, 3H), ^13^C NMR (126 MHz, Chloroform-d) δ 174.27, 51.37, 34.08, 31.83, 29.38, 29.23, 29.23, 29.13, 24.94, 22.63, 14.05.

### 2.2. Electrophysiological Assays

#### 2.2.1. Patch-Clamp Recordings

Patch-clamp recordings were performed essentially as described by Larson and colleagues [13]. HEK-293 cells transfected with *An. gambiae* Kv2.1 channel gene were cultured in DMEM/F12 medium containing 10% FBS, 100 µg/mL hygromycin and 15 µg/mL blasticidine. Expression of the AgKv2.1 channel was induced by 10 µg/mL tetracycline for 24 h. The whole-cell patch-clamp was done with standard extracellular solution (in mM, 140 NaCl, 5 KCl, 2 CaCl_2_, 2 MgCl_2_, 10 glucose, 10 HEPES, pH 7.4) and intracellular solution (in mM, 10 NaCl, 120 KCl, 1 CaCl_2_, 2 MgCl_2_, 10 EGTA, 10 glucose, 10 HEPES, pH 7.2). The resting potential was held at −80 mV. Currents were evoked by stepping the membrane potential from −100 to +100 mV in 20 mV increments. Current block was determined every 3 min at increasing concentrations of individual compounds, from 10 nM to 100 µM. Stock solutions were made by dissolving the putative insecticides in DMSO, followed by dilution to the desired concentration in extracellular buffer. Current signals were recorded and analyzed by using Axon 200B amplifier and pClamp10.7 software. IC_50_ values and slopes were calculated by using GraphPad Prism 7 (GraphPad Software, San Diego, CA, USA).

#### 2.2.2. Drosophila Melanogaster Central Nervous System Recordings

Central nervous system (CNS) preparations were made from isolated ventral ganglion of third-instar larvae of *D. melanogaster* Oregon-R wild type strain. This model relies on the extracellular recording of the spontaneous synchronized bursting activity of the motor neuron fibers located in the abdominal nerves exiting the ventral ganglion [29]. Spikes were converted to a rate (in Hz) using the threshold counting function by LabChart 7 software (ADInstruments, Colorado Springs, CO, USA). Spike counting threshold was set above background noise (set when no peripheral nerves were attached to the suction electrode), and a baseline frequency was established for 10 min. The CNS preparation was put in a saline bath (in mM, 157 NaCl, 7 CaCl2, 3 KCl, 4 HEPES, pH 7.2 in distillated water). Tested compounds were added individually to the saline bath (0.1% DMSO final). The pre-treatment baseline and drug-induced nerve firing rates (after treatment) were each averaged for 3-min periods, over the course of the 30-min recording.

### 2.3. Insects

Mosquitoes were reared to adulthood from *Anopheles gambiae* eggs collected from an insecticide-susceptible G3 (MRA112) strain obtained from the CDC (Atlanta, GA, USA). Eggs were placed in a water tray and incubated at 28 °C, 75% relative humidity, light/dark cycle 12 h/12 h. Larvae were fed ground fish flakes (Tetra Holding, Blacksburg, VA, USA), and reared to adulthood. Upon test, adult mosquitoes were kept in small cups closed with a mesh and fed with 10% sucrose solution on cotton balls. The susceptible Oregon-R strain of *Drosophila melanogaster* was reared as previously described [29] at 21 °C, and provided with artificial medium purchased from Carolina Biological Supply (Burlington, NC, USA).

### 2.4. Whole Insect Toxicity Assays

#### 2.4.1. Larval Paralysis Assays

Paralytic effect was assessed as previously described on intact larvae [13] and on headless larvae [30]. Heads were removed from fourth-instar larvae of *Anopheles gambiae* using two forceps, by pinching at the neck. Intact or headless larvae were then placed in petri dishes containing mosquito larval saline (in mM; 154 NaCl, 2.7 KCl, 1.4 CaCl_2_, 5 NaHCO_3_, 4 HEPES, pH 6.9). Lethality towards intact larvae was determined at 24 h, and motility in headless larvae was assessed every hour up to 5 h [30]. Larvae were separated into categories, either “active” or “paralyzed/dead” and a 5 h paralytic concentration (PC_50_) or 24 h LC_50_ was determined. Positive control was L-aspartic acid at 10 ppm, which typically paralyses ca. 50% of headless larvae in 5 h. Lipophilic compounds were diluted in 100% ethanol, which was used at 0.5% final concentration of vehicle. PC_50_ and LC_50_ values were calculated by using GraphPad Prism 7 and IBM SPSS 23.

#### 2.4.2. Topical Application

**Figure 1:**
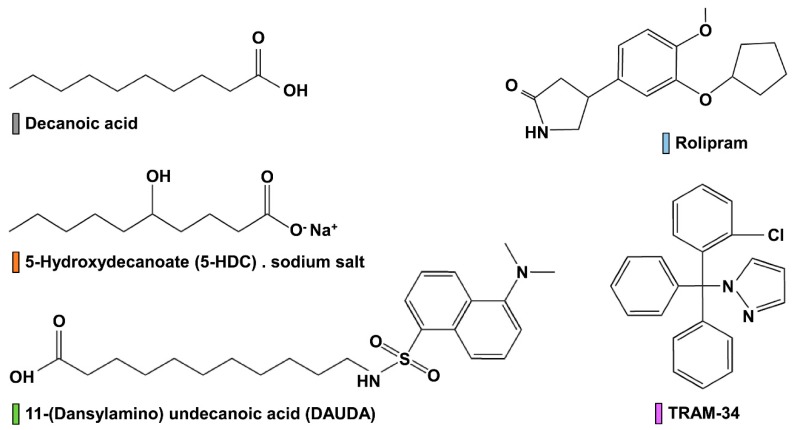
Chemical structures of commercially available compounds. Colored bars next to each compound name match the corresponding symbol color in the Figures.

Adult mosquitoes (female) of *Anopheles gambiae* were treated with 200 nL of the compounds shown in Figure 1 (solvent: 100% ethanol). Knockdown at 1 and 4 h (uncontrollable walking/flight or complete inhibition of flight), and mortality at 24 h (no visible movement) were determined. LD_50_ values and slopes were calculated by using IBM SPSS 23. Knockdown and mortality were observed and corrected with Abbott’s formula [31]. Individual compounds were also tested as a co-application with permethrin. The LD_15_ value of each compound was calculated from the regular topical application described just above. This fixed dose was co-applied with a permethrin dose range. Same as above, knockdown at 1 h and 4 h, and mortality at 24 h were observed; KD_50_ and LD_50_ values of permethrin alone (positive control) and co-applied with each compound were determined.

### 2.5. Data Analysis

For patch-clamp experiments, headless larvae assays and topical applications, IC_50_, PC_50_ and LD_50_ values were determined respectively, either through probit analyses or by non-linear regression. These models give a 95% confidence interval (95% CI) and one simple yet efficient way to compare mean values within a respective experiment is to note any overlap of 95% CI. Wheeler and colleagues [32] determined that this type of analysis can generate more type-2 errors (accepting that two values are not different when they actually are) only when there was a slight overlap of 95% CI. In this present report, we did not encounter this situation: we either had strong overlaps or non-overlapping 95% CI. For CNS recordings, we used One-way ANOVA and Dunnett’s post-hoc tests to compare groups relative to control. All data analyses were done using IBM SPSS 25 and GraphPad Prism 7.

## 3. Results

### 3.1. Electrophysiological Assays

#### 3.1.1. Patch Clamp Recordings

**Figure 2:**
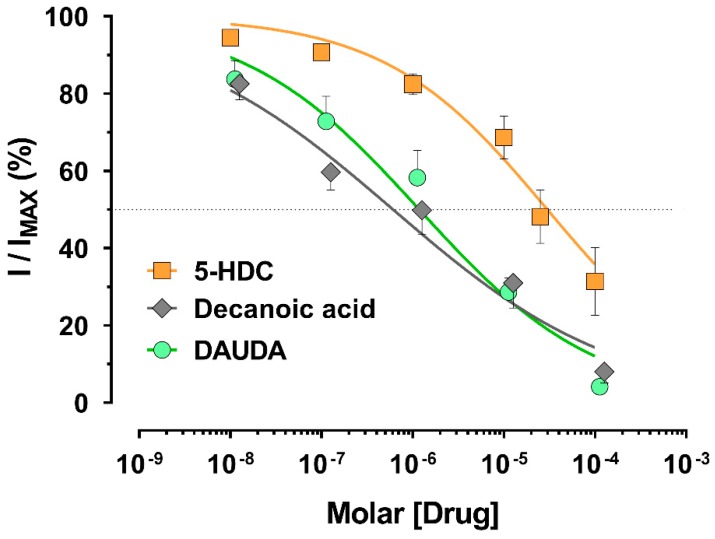
Concentration-response curves of compounds on AgKv2.1 channels. Patch-clamp whole-cell recordings were performed on HEK-293 cells expressing AgKv2.1 channels. Current inhibition was determined at +60 mV. Current amplitude for each concentration of compounds was related to a control recording with extracellular solution without drug. Compounds were diluted in 0.1% DMSO final. The concentration that inhibited 50% of the control current amplitude (IC_50_) was determined and reported in Table 1. The dotted line is the 50% inhibition level.

We tested the different compounds as potential *An. gambiae* AgKv2.1 channel blockers. Although rolipram and TRAM-34 were inactive at 3 mM and 10 mM, respectively; 5-HDC, decanoic acid, and DAUDA were effective inhibitors of voltage-gated potassium currents, but with different potencies (Figure 2). The concentration inhibiting 50% of the control current (IC_50_) was calculated and reported in Table 1. Decanoic acid and DAUDA (IC_50_ = 0.6 and 1.2 µM, respectively) inhibited the potassium current in a similar range; 5-HDC, in contrast, had a significantly higher IC_50_ value (30 µM) compared to the two other compounds (cf. Table 1, non-overlapping 95% CI), making the 5-HDC a less potent K^+^ channel blocker.

#### 3.1.2. CNS Recordings

**Figure 3:**
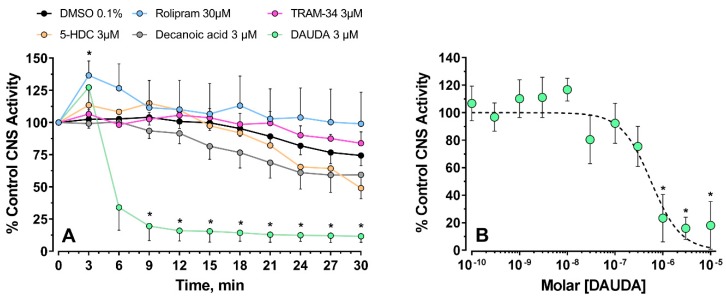
Recording the activity of *Drosophila* CNS under pharmacological screen. (**A**) CNS activity was recorded for 30 min and the firing rate expressed relative to the control (pretreatment) observed at the start of the recording. Asterisks indicate significant difference from DMSO controls (* *p* < 0.05). (**B**), Non-linear fit of log-transformed concentrations of 11-dansylamino undecanoic acid (DAUDA), showing a significant inhibition of the CNS activity at 1 µM and higher concentrations (* *p* < 0.004 or less). In all cases, symbols are means ± SEM.

When the compounds were tested on Drosophila CNS preparations, they had different effects and potencies. Preparations were challenged with compounds at 3 µM, except rolipram, which was applied at 30 µM (Figure 3). Under these conditions, rolipram significantly increased firing after 3 min incubation that declined in intensity thereafter, whereas the other materials had varying levels of inhibition. At 9 min post-treatment, only DAUDA significantly reduced CNS activity (One-way ANOVA, F _(5, 40)_ = 3.492, *p* = 0.0103, Dunnett’s comparison CTRL/DAUDA, *p* = 0.0016). DAUDA was tested further with a range of concentrations, and the CNS activity significantly decreased at concentrations of 1 µM and higher (Figure 3B). One-way ANOVA gave, F _(11, 67)_ = 5.55, *p* < 0.001; Dunnett’s post-test, CTRL/DAUDA 1 µM, *p* < 0.001, CTRL/DAUDA 3 µM, *p* = 0.004, CTRL/DAUDA 10 µM, *p* < 0.001). In a similar fashion to the patch-clamp experiment, DAUDA blocked Drosophila CNS activity with IC_50_ = 0.57 [0.30–1.45] µM. 5-HDC was also studied at additional concentrations, and at 10 and 30 µM, no significant change in firing rate was observed (data not shown).

### 3.2. Larval Paralysis

To assess in vivo toxicity, we first screened the different fatty acids and potential K^+^ channel blockers on intact *An. gambiae* G3 larvae (Table 1). At 250 ppm, rolipram and TRAM-34 paralyzed less than 10% of the tested larvae, and because of this result, and the previous absence of effect on CNS activity, rolipram and TRAM-34 were not tested in the headless larvae assay. The other compounds paralyzed between 14 to 30% of the intact larvae (Table 1), and a paralytic concentration that affects 50% of the tested larvae, PC_50_, was determined. Decanoic acid and DAUDA paralyzed the larvae at a similar concentration range (PC_50_ = 32 and 21 ppm, respectively, with overlapping 95% CI). In contrast, 5-HDC had significantly higher PC_50_ value (173 ppm) compared to the two other compounds (Table 1, non-overlapping 95% CI), again making 5-HDC a less effective compound for paralyzing headless larvae.

### 3.3. Lethality by Topical Application

All the compounds of interest were tested topically on *An. gambiae* G3 adult mosquitoes. At 1 h after treatment (Table 2), decanoic acid was the most potent compound to knockdown mosquitoes (440 ng/mg), followed by 5-HDC and TRAM-34 (around 700 ng/mg), and then rolipram (1.2 µg/mg), with DAUDA (1.9 µg/mg) being the least toxic, and different from the other fatty acids (Table 2, non-overlapping 95% CI). After 4 h, decanoic acid was still the most potent to knockdown mosquitoes (330 ng/mg, cf. Table 2). decanoic acid was significantly different from the other fatty acids, as well as rolipram and TRAM-34, whose KD_50_ values were between 1 and 2 µg/mg (non-overlapping 95% CI, Table 2). When assessing mortality at 24 h, only decanoic acid had an LD_50_ value lower than 1.0 µg/mg (Table 2). Decanoic acid was different from DAUDA (Table 1, LD_50_ = 8.9 µg/mg, non-overlapping 95% CI), but 5-HDC, which was intermediate in toxicity, was not different from decanoic acid or DAUDA (Table 1, LD_50_ = 2.6 µg/mg, overlapping 95% CI). Furthermore, since rolipram and TRAM-34 killed less than 50% of the mosquitoes tested at the highest soluble concentration (25 mg/mL, equivalent to 5 µg/mosquito), the given LD_50_ values are only estimates and the 95% confidence intervals for each compound are very broad (Table 1).

### 3.4. Evaluation of Permethrin Synergism

All the compounds were individually tested with permethrin co-application. From the probit analyses done previously, doses corresponding to the LD_15_ of each compound were determined and used in the synergism studies (Table 3). When compared to the effect of permethrin alone, decanoic acid and 5-HDC both increased the knockdown effect of permethrin 1 h after application by non-overlapping 95% CI. DAUDA, when co-applied with permethrin, had a lower KD_50_ value than permethrin alone, but it was not significantly different from it nor decanoic acid and 5-HDC. After 4 h, only the co-application of DAUDA significantly increased the action of permethrin (Table 3, KD_50_ (1 h). Co-applied decanoic acid and 5-HDC, while having slightly lower KD_50_ values than permethrin alone at 4 h, were not significantly different from it. After 24 h, when assessing mortality, no co-applied compounds had significantly different LD_50_ values than permethrin alone, although 5-HDC had an LD_50_ value significantly less than co-applied rolipram and DAUDA. TRAM-34 and rolipram were not significantly different from permethrin alone at any time point, and they had slightly higher KD_50_ and LD_50_ values than permethrin by itself (Table 3).

**Figure 4:**
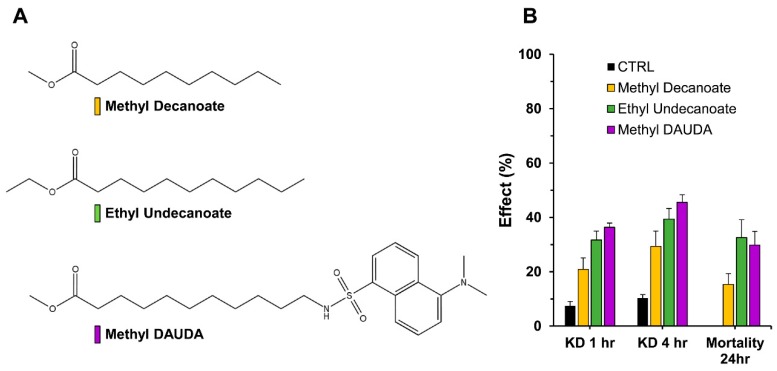
Fatty acid esters and topical application. (**A**) Structures of methyl decanoate, ethyl undecanoate, and methyl DAUDA. These three compounds were topically applied to *An. gambiae* adult females. (**B**) At a screening dose of 1 µg per *An. gambiae* mosquito, percentages of knockdown and mortality are reported.

It was previously suggested that DAUDA could bind to the KcsA channel while closed [17], meaning it could reach the channel pore cavity through the lipid bilayer of the cell. But results from intact larvae assays and topical applications showed that the cuticle might impede the delivery of the compounds, thus decreasing the efficacy of their potential cellular effects. To examine this issue further and try to alleviate this barrier, structural modifications of the compounds were attempted that would allow cuticle penetration, opening new leads for fatty acid insecticides. Accordingly, three fatty acid esters were prepared; namely, methyl DAUDA, methyl decanoate, and ethyl undecanoate. The hypothesis was that esterification would augment penetration, and the appended alkyl group would be removed by carboxylesterases within the insect. As showed in Figure 4, in similar fashion as the parent FAs, these compounds showed some efficacy for knockdown, and less for mortality. Knockdown approached, but never exceeded 50% in 4 h, with mortality of no more than 30%. Also, a clear rank order of effect was present across all KD and LD measurements: methyl decanoate < ethyl undecanoate < methyl DAUDA (Figure 4). Overall, this level of activity was generally less than the free acid forms (Table 2).

## 4. Discussion

The tested fatty acids were effective blockers of Kv2.1 channels in patch-clamp whole-cell recordings that depended upon their chemical structure with a rank order of potency of decanoic acid = DAUDA, > 5-HDC > TRAM-34 and rolipram (Figure 2). Previously reported catechol compounds 4-*tert-*octylcatechol, 3-hexylcatechol, and 3-(3-methylbutan-2yl) catechol had IC_50_ values of 44, 53, and 26 µM, respectively [13]. For comparison, decanoic acid and DAUDA had more than 10-fold lower IC_50_ values (Table 1), suggesting higher affinity for blocking potassium channel activity. Additionally, 5-HDC (IC_50_ = 30 µM) was the least potent compound acting on Kv channels, but it had a similar potency to these reported catechols. Finally, our reported compounds all showed better blocking potency compared to established *Ag*Kv2.1 channel blockers, 4-aminopyridine and tetraethylammonium, which had IC_50_ values of 5.6 and 1.5 mM, respectively on the *Ag*Kv2 channel expressed in HEK-293 cells [13].

In a similar fashion, the effects of tested compounds on headless larvae show a comparable trend to that observed with whole-cell recordings (Table 1). The fatty acids decanoic acid and DAUDA effectively blocked larval movements (PC_50_ values between 21 and 32 ppm), while 5-HDC was revealed to be 5- to 8-fold less active. Our results are again in line with previous results reported for catechols (PC_50_ values between 6.6 and 45 ppm) [13]. Also, 5-HDC showed a paralytic efficacy very similar to tetraethylammonium (PC_50_ of 173 ppm for both), but lower activity on intact larvae, probably related to slow passage through the cuticular barrier.

It is interesting to observe the striking difference of results between decanoic acid and 5-HDC in every assay (Table 1). Decanoic acid always yields better efficacies compared to 5-HDC, yet the only structural difference resides in the hydroxyl group in position 5 of the carbon chain for 5-HDC (Figure 1). If we consider the carbon chain to fit in lumen of the Kv channel pore, can this additional hydroxyl group generate a steric hindrance that would reduce 5-HDC binding within the pore? It has been shown that tetraethylammonium can bind to the prokaryotic potassium channel KcsA [33] and with numerous eukaryotic channels, including *Ag*Kv2.1 [13]. Additionally, Smithers and colleagues [17] determined that fatty acids bound within the central cavity of KcsA, and they used DAUDA and other fatty acids of various lengths to determine that the binding increased with number of carbons in the chain, from C_14_ to C_20_ [17]. The presence of double bonds had little effect on binding and dissociation constants [17]; but there is no mention of the effect of chemical substitution within the carbon chain. Here, we report and confirm for the first time the effect of DAUDA on a eukaryotic potassium channel, *Ag*Kv2.1, and that the presence of a hydroxyl group in the carbon chain (decanoic acid/5-HDC) results in a 50-fold difference in channel blocking affinity (Table 1). Such a result was predicted by Boland and Drzewiecki [11] when they discussed FA mechanistic actions on Kv channels: they suggested that FA need sufficient membrane solubility in order to reach the channel pore, and hypothesized that making a FA less soluble in the membrane by adding a hydrophilic group would affect its ability to modulate channel function.

Fatty acids as contact insecticides have been suggested and tested almost a century ago [34] and since then, the literature about their mosquitocidal effect is sparse. Decanoic acid is a natural FA found in coconut oil, palm kernel oil, and goat’s milk [35]. Integrated pest management is looking toward natural products and other biopesticides, in better accordance with legislative framework [27,36]. Along these lines, we attempted to increase lethal activity of decanoic acid and other FAs by alkylating the acid group in the expectation that it would increase cuticular permeability. Unfortunately, the compounds were even less toxic than the free acids. The reasons behind the failure of this effort are unclear, but perhaps penetration is not positively affected or in vivo bioactivation by esterases does not occur to an appreciable extent.

Another way to use FAs to increase insect toxicity was examined through synergistic effects with co-applied compounds. It has been shown that permethrin can be synergized by essential oils [37] and that potassium channel blockers can increase its toxicity [38]. In the present study, FA co-applied with permethrin yielded differential toxicity rates (Table 3): only 5-HDC slightly decreased the LD_50_ value of permethrin, while DAUDA and decanoic acid had no visible effect on mortality. Permethrin synergistic action on mosquitoes has been shown with different compounds such as propoxur or flonicamid, acting on different targets than Kv channels [26,39]. Moreover, piperonyl butoxide (PBO), a known synergistic agent to many pesticide compounds [40,41], has also been tested in co-application with decanoic acid or 5-HDC (see Supplementary Figure S1 and Table S1). Unfortunately, no significant effect was observed on mosquitos’ knockdown or mortality. Our results suggest that neither permethrin nor PBO have any potent synergistic effect with FA compounds. This could be due to co-applied doses that were not concentrated enough, or perhaps due the passage of the FA compounds through the cuticle, which may prevent or reduce the synergistic effect with permethrin or PBO. In order to facilitate the passage of FAs through the cuticle and membranes and enhance synergistic effects, further works will focus on the carrier solvent to strengthen the link from cellular-level effects to the toxicity at the behavioral level.

## 5. Conclusions

In conclusion, fatty acids have blocking actions on Kv channels and insect CNS firing, and they may represent a novel lead for mosquitocidal compounds, but only if cuticle penetration can be addressed. On the other hand, the two compounds TRAM-34 and rolipram, although related to potassium channel function, had no insecticidal effect on larval or adult mosquitoes.

## Figures and Tables

**Table 1:** Activity of each compound across different assays. Calculated values and 95% CI [in brackets] are given for *Ag*Kv2 channel inhibition (Patch-Clamp: IC_50_), paralysis (Headless Larvae: PC_50_, and Intact Larvae: percentage) and lethality (Topical Application: LD_50_ to adult females). Statistical significance was assessed according to non-overlap of 95% CI, where means within a column not labeled by the same letter are different (*p* < 0.05).

Compounds	Patch Clamp	Headless Larvae	Intact Larvae	Topical Application
	IC_50_	PC_50_	% Paralysis	LD_50_
	(µM)	(ppm)	(250 ppm)	(µg/mg BW)
**Rolipram**	N.A.	N.A.	3.0 a	20.9 a
			[0–8.9]	[4.2–1.3 × 10^5^]
**TRAM-34**	N.A.	N.A.	6.4 a	15.6 a
			[0.1–12.7]	[2.9–5.0 × 10^6^]
**5-HDC**	30 a	173 a	13.8 ab	2.6 ab
	[19–57]	[90–480]	[5.6–22.0]	[1.6–6.3]
**Decanoic acid**	0.61 b	32 b	29.5 b	0.8 b
	[0.3–1.2]	[26–42]	[21.5–37.6]	[0.3–2.9]
**DAUDA**	1.18 b	21 b	23.2 b	8.9 ab
	[0.6–2.2]	[9–83]	[18.9–27.5]	[3.5–65]

**Table 2:** Activity of each compound topically applied to adult female *An. gambiae*. KD_50_ and LD_50_ values with [95% CIs] are in µg/mg of adult body weight. Values for response slope (±SEM) are also shown. Responses were compared to each other according to the 95% CI of the KD_50_ or LD_50_. For each time point (column), different letters mean non-overlapping 95% CI, and KD_50_ or LD_50_ values not labeled by the same letter are different (*p* < 0.05). NB: for rolipram and TRAM-34, mortality failed to reach 50%, so LD_50_ values were extrapolated values from the probit analyses and indicated in italics.

Compounds	1 h		4 h		24 h	
	KD_50_	Slope	KD_50_	Slope	LD_50_	Slope
**Rolipram**	1.18 ab	0.96	1.93 a	0.7	20.9 a	0.9
	[0.72–2.86]	(0.07)	[0.91–12.3]	(0.06)	*[4.23–1.3 × 10^5^]*	(0.11)
**TRAM-34**	0.74 a	1.5	0.81 a	0.94	15.6 a	0.75
	[0.54–1.10]	(0.07)	[0.55–1.43]	(0.06)	*[2.86–5.0 × 10^6^]*	(0.08)
**5-HDC**	0.71 a	1.44	0.91 a	1.28	2.62 ab	1.29
	[0.58–0.91]	(0.07)	[0.66–1.41]	(0.07)	[1.60–6.28]	(0.09)
**Decanoic acid**	0.44 a	1.41	0.33 b	1.56	0.80 b	1.77
	[0.22–1.01]	(0.07)	[0.22–0.52]	(0.08)	[0.29–2.92]	(0.09)
**DAUDA**	1.91 b	1.1	1.7 a	0.71	8.90 a	0.84
	[1.23–3.35]	(0.06)	[0.84–5.03]	(0.05)	[3.82–64.9]	(0.06)

**Table 3:** Synergistic activity of each compound co-applied with permethrin. KD_50_ and LD_50_ values with [95% CIs] are given in µg/mg of adult body weight, and doses co-applied correspond to the 24 h LD_15_ of each compound. Values for response slope (±SEM) are also shown. Responses were compared to each other according to the 95% CI of the KD_50_ or LD_50_. For each time point (column), different letters mean non-overlapping 95% CI, and KD_50_ or LD_50_ values not labeled by the same letter are different (*p* < 0.05).

Compound	Synergist	1 h		4 h		24 h	
	Dose (ng)	KD_50_	Slope	KD_50_	Slope	LD_50_	Slope
**Permethrin**	0	0.08 a	3	0.08 a	2.22	0.10 ab	2.36
		[0.07–0.09]	(0.1)	[0.06–0.10]	(0.04)	[0.08–0.12]	(0.08)
**+ Rolipram**	300	0.08 a	2.67	0.11 a	1.5	0.15 a	2.1
		[0.07–0.10]	(0.14)	[0.08–0.16]	(0.12)	[0.11–0.21]	(0.13)
**+ TRAM-34**	300	0.11 a	2.03	0.09 ab	1.49	0.10 ab	2.14
		[0.08–0.13]	(0.13)	[0.03–0.15]	(0.11)	[0.05–0.18]	(0.13)
**+ 5-HDC**	300	0.03 b	1.99	0.06 ab	1.67	0.06 b	2.16
		[0.02–0.04]	(0.15)	[0.02–0.09]	(0.12)	[0.03–0.09]	(0.14)
**+ Decanoic acid**	250	0.03 b	1.92	0.04 ab	1.06	0.14 ab	2.17
		[0.02–0.04]	(0.09)	[0.02–0.07]	(0.07)	[0.07–0.48]	(0.11)
**+ DAUDA**	500	0.06 ab	1.11	0.03 b	1.04	0.15 a	1.46
		[0.03–0.09]	(0.06)	[0.02–0.04]	(0.06)	[0.10–0.29]	(0.06)

## Supplementary Materials

The following are available online at http://www.mdpi.com/2075-4450/9/4/155/s1.
