# Supplementary material for: Fatty Acid and Related Potassium Kv2 Channel Blockers: Toxicity and Physiological Actions on Mosquitoes
Source: Insects. 2018 Nov 1;9(4):155. doi: 10.3390/insects9040155 (PMC6315728; doi:10.3390/insects9040155)
Supplement: Supplementary file 1 [file insects-09-00155-s001.pdf]

FATTY ACID AND RELATED POTASSIUM KV2 CHANNEL BLOCKERS: TOXICITY AND PHYSIOLOGICAL ACTIONS ON MOSQUITOES.

FABIEN DÉMARES [1,*], QUENTIN COQUEREL [1], GARY RICHOUX [1], KENNETH LINTHICUM [2] AND JEFFREY BLOOMQUIST [1,*]

[1] Neurotoxicology Laboratory, Entomology and Nematology Department, Emerging Pathogens Institute, University of Florida, Gainesville, FL, USA.

[2] USDA, ARS, Center for Medical, Agricultural and Veterinary Entomology, Gainesville, FL, USA.

***** Correspondence: fabien.demares@ufl.edu; jbquist@ufl.edu; Tel.: +1 (352) 294-5166.

Supplementary Materials

Figure S1. Co-application of piperonyl butoxide (PBO) with fatty acid compounds.

Table S1. Comparison of 5-HDC alone and 5-HDC/PBO co-application on *Anopheles gambiae* G3, with probit slopes and KD_{50}/LD_{50} values.

Figure S1.

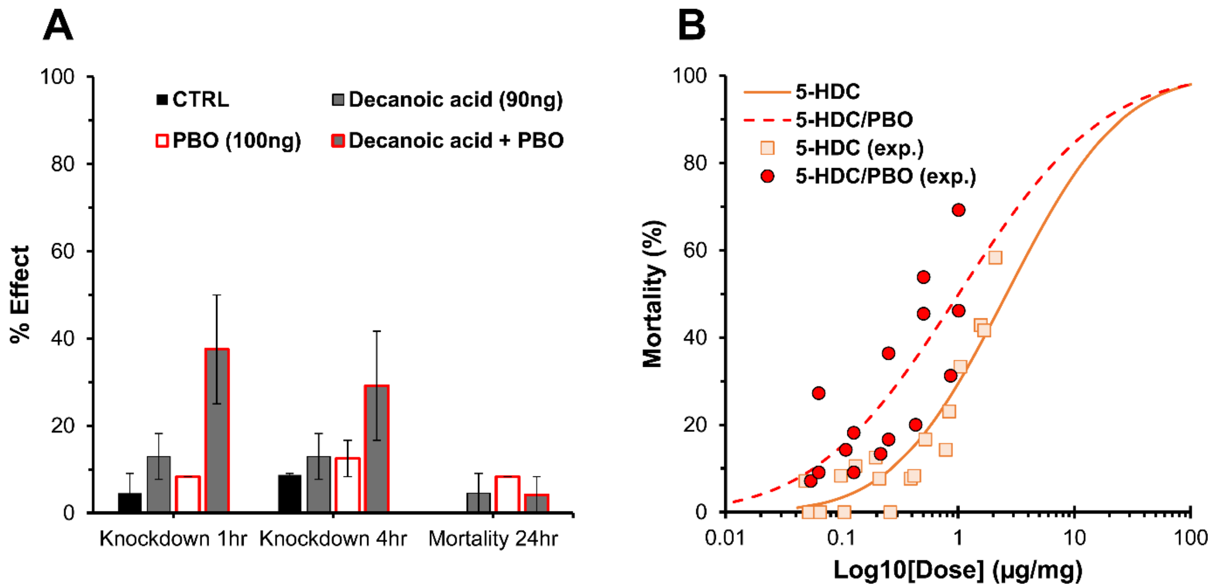

Figure S1. Co-application of piperonyl butoxide (PBO) with fatty acid compounds. (**A**) Decanoic acid was tested for one dose (LD_{15} = 90 ng) and co-applied with 100 ng of PBO (which correspond to LD_{15} as well). Despite a visible knockdown effect (not statistically significant) of co-applied decanoic acid and PBO after 1 and 4 hours, there was no significant synergistic effect on mortality at 24 hours. (**B**) For 5-HDC, the probit analyses (curves) were calculated from a range of tested doses (colored symbols, experimental values). The slopes and LD_{50} values have been determined, and are reported in Table S1. For 5-HDC alone, LD_{50} = 2.6 µg; for co-applied 5-HDC/PBO, LD_{50} = 1.0 µg. But the 95% confidence intervals overlap extensively, which means no significant difference between topical application with or without PBO.

Table S1. Comparison of 5-HDC alone and 5-HDC/PBO co-application on *Anopheles gambiae* G3, with probit slopes and KD_{50}/LD_{50} values. KD and LD values are in µg/mg of body weight.

	1 hour		4 hours		24 hours	
	**Slope (SE)**	**KD_{50} [CI95%]**	**Slope (SE)**	**KD_{50} [CI95%]**	**Slope (SE)**	**LD_{50} [CI95%]**
**5-HDC**	1.44 (0.07)	0.71 [0.58 - 0.91]	1.28 (0.07)	0.91 [0.66 - 1.41]	1.29 (0.09)	2.62 [1.6 - 6.26]
**5-HDC + PBO**	1.03 (0.08)	0.23 [0.15 - 0.37]	0.71 (0.08)	0.27 [0.15 - 0.55]	1.03 (0.09)	1.01 [0.56 - 4.35]
